# Structural, physicochemical and dynamic features conserved within the aerolysin pore-forming toxin family

**DOI:** 10.1038/s41598-017-13714-4

**Published:** 2017-10-24

**Authors:** Nuria Cirauqui, Luciano A. Abriata, F. Gisou van der Goot, Matteo Dal Peraro

**Affiliations:** 1 Institute of Bioengineering, School of Life Sciences, École Polytechnique Fédérale de Lausanne (EPFL), 1015 Lausanne, Switzerland; 2 Department of Pharmaceutical Biotechnology, Universidade Federal do Rio de Janeiro, 21941-902 Rio de Janeiro, Brazil; 3 Global Health Institute, School of Life Sciences, École Polytechnique Fédérale de Lausanne (EPFL), 1015 Lausanne, Switzerland

## Abstract

Aerolysin is the founding member of a major class of β-pore-forming toxins (β-PFTs) found throughout all kingdoms of life. PFTs are cytotoxic proteins produced as soluble monomers, which oligomerize at the membrane of target host cells forming pores that may lead to osmotic lysis and cell death. Besides their role in microbial infection, they have become interesting for their potential as biotechnological sensors and delivery systems. Using an approach that integrates bioinformatics with molecular modeling and simulation, we looked for conserved features across this large toxin family. The cell surface-binding domains present high variability within the family to provide membrane receptor specificity. On the contrary, the novel concentric double β-barrel structure found in aerolysin is highly conserved in terms of sequence, structure and conformational dynamics, which likely contribute to preserve a common transition mechanism from the prepore to the mature pore within the family.Our results point to the key role of several amino acids in the conformational changes needed for oligomerization and further pore formation, such as Y221, W227, P248, Q263 and L277, which we propose are involved in the release of the stem loop and the two adjacent β-strands to form the transmembrane β-barrel.

## Introduction

Pore-forming toxins (PFTs) are cytotoxic proteins produced by a variety of organisms. Apart from their virulence, they are emerging as interesting assemblies for their potential as biotechnological sensors and delivery systems^1–4^.

**Figure 1:**
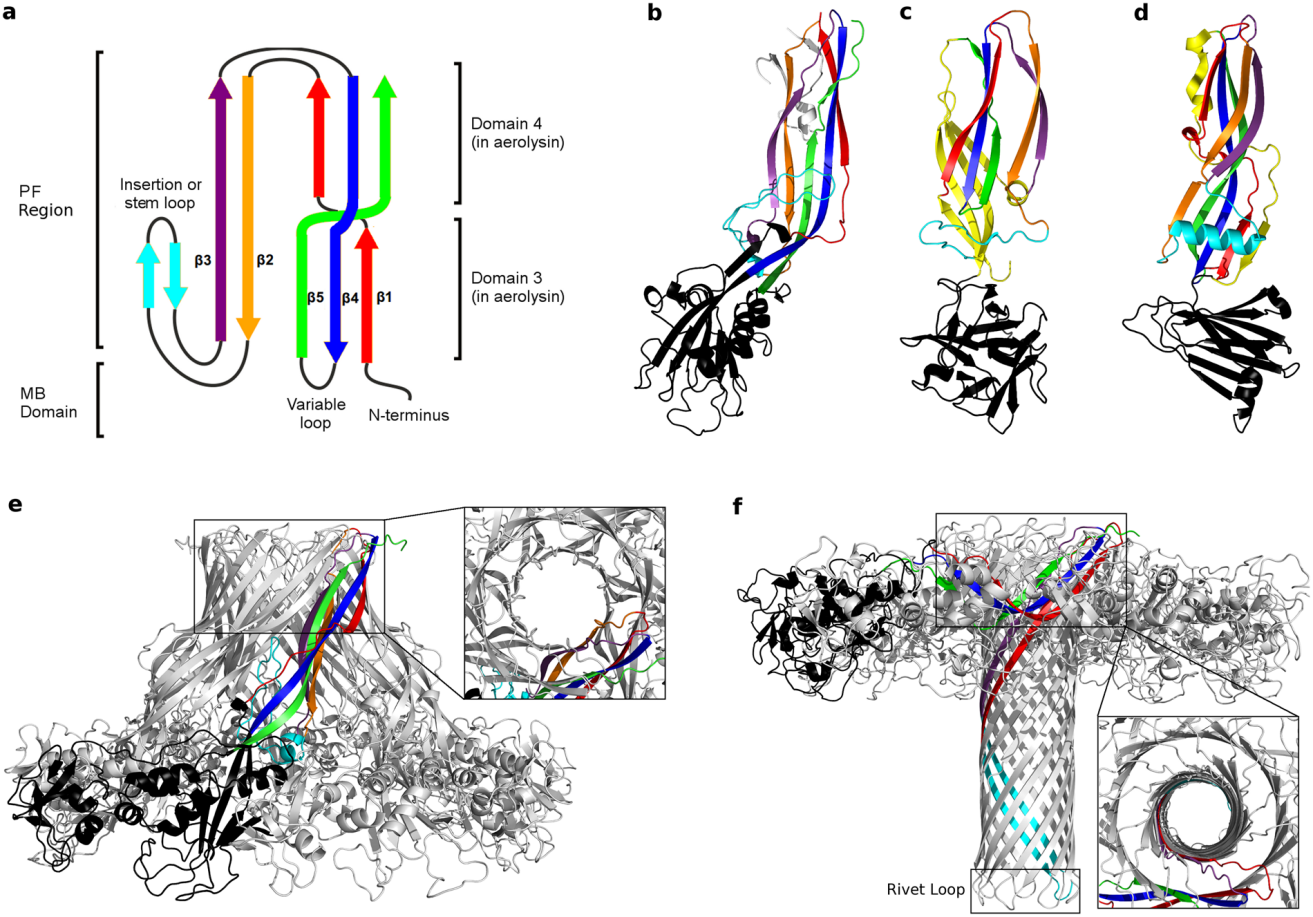
Toxin structure and pore architecture in the aerolysin-like family. (**a**) Simplified topology scheme of the common core of aerolysin-like β-PFTs, showing the MB and the PF regions. Figure adapted from Szczesny *et al*.^6^. (**b**) Aerolysin soluble monomer structure (PDB entry 1PRE)^19^, with the same color scheme as **a** for the PF region, the MB domain shown in black and the CTP in grey. Domain 1 has been deleted to help visualization. (**c**) Lysenin soluble monomer structure (PDB entry 3ZX7)^10^, with the same color scheme as **b**. The four extra β-sheets are colored in yellow. (**d**) CPE soluble monomer structure (PDB entry 2XH6)^17^, with the same color scheme as (**c**). (**e**) Aerolysin prepore structure (PDB entry 5JZH)^20^, with one protomer shown in colors. In the inset a close-up of a partially formed DBB is shown from a view perpendicular to the membrane. (**f**) Aerolysin pore structure (model based on quasi-pore structure PDB entry 5JZT)^20^, showing at the bottom the rivet loop of the TM barrel, and in the close-up a totally formed DBB.

Aerolysin, which was the first β-PFT with solved monomer structure, is the founding member of a major class of PFTs found throughout all kingdoms of life^5,6^. Five other 5 aerolysin-like toxin structures were also solved in their soluble form, namely the epsilon-toxin (ETX) from the anaerobic Gram-positive *Clostridium perfringens* ^7^, parasporin-2 from *Bacillus thuringiensis* ^8^, *Laetiporus sulphureus* lectin (LSL) from the mushroom *Laetiporus sulphureus* ^9^, lysenin from the earthworm *Eisenia fetida* ^10^, and Dln1 from *Danio regio* ^11^. All these aerolysin-like proteins share a similar monomeric architecture, with a variable membrane-binding (MB) domain and a structurally conserved pore-forming (PF) region, presenting five β-strands with an insertion loop (stem loop (SL)) between strands β2 and β3 (Fig. 1a)^6^. The MB domain is formed by the N-terminus and a variable loop between strands β4 and β5^6^. The information known about the activation processes and oligomer stoichiometry of these proteins have been already reviewed^3,12^. Their similar monomer fold suggests similar pore forming mechanisms, which has been recently supported by the similarity between the lysenin and the aerolysin pores, even though lysenin diverges the most from other toxins of the family in both the orientation of the stem loop (together with strands β2-β3) and the existence of four extra β-sheets (Fig. 1c)^10,13,14^. Moreover, the HA3 subcomponent of *Clostridium botulinum* type C progenitor toxin (HA3)^15,16^ and *Clostridium perfringens* enterotoxin (CPE)^17,18^, show the same main architecture of aerolysin at the PF region, but present an alpha helical stem loop and an extra β-strand (Fig. 1d).

Recently, cryo-EM structures at near-atomic resolution have been reported for aerolysin mutants arrested at different stages of pore formation, namely the prepore^20^ (PDB entry 5JZH), post-prepore^20^ (PDB entry 5JZW), and quasi-pore states^20^ (PDB entry 5JZW), together with a low resolution pore conformation^20^ (PDB entry 5JZT), making this toxin the best characterized among all β-PFTs. Aerolysin binds to its target host cell via GPI-anchored proteins^21^. Upon proteolytic cleavage of the C-terminal peptide (CTP, Fig. 1b), aerolysin is activated and oligomerizes into a prepore state showing a novel fold, here named double β-barrel (DBB), constituted by two concentric barrels formed by strands β2-β3 (inner β-barrel) and strands β1-β4-β5 (outer β-barrel), held together mainly by hydrophobic interactions^20^ (Fig. 1e,f). Afterwards, the stem loop located in domain 3, together with the successive two β-strands (β2 and β3, Fig. 1a), extends out of its pocket and move towards the membrane creating a transmembrane (TM) β-barrel (Fig. 1f). Subsequently, the protein collapses vertically bringing both concentric β-barrels towards the target membrane in a piston-like movement^20^. The recently solved structures of lysenin by both cryo-EM and X-ray crystallography, while showing the same general arrangement of the β-strands, present a much shorter collapse along with a different stoichiometry^13,14,20^. This information has been reviewed recently^22^.

In order to expand our understanding of the pore forming mechanism within the aerolysin-like family, we performed an exhaustive analysis integrating bioinformatics and molecular modeling, which allowed us to reveal the key role of some amino acids, as well as conserved motions localized at the DBB region. Altogether, our results suggest that the DBB is not only conserved within the family, but that the conformational rearrangements required to fold it may act as driving forces for oligomerization and further pore-formation. Besides these conserved features shared across the family, lateral domains surrounding the central DBB structure are instead very variable, possibly to confer specificity for membrane receptors, producing thus a high degree of modularity for this PFT family.

## Results

### Overall conservation and variability in aerolysin-like PFTs

PFTs are proteins with a conserved structural fold despite their low sequence identity, which thwarts their classification into protein families. As shown by Szczesny *et al*. through sequence searches using the core PF region as query, a large number of proteins containing aerolysin domains are distributed throughout all kingdoms of life, including higher eukaryotes^6^. In the set of reported proteins, the aerolysin-like domain was accompanied by at least one of 8 types of domains specialized in binding different molecules, located N- and/or C-terminally to the aerolysin domain. Moreover, when extending this analysis, we found that proteins containing the aerolysin core connected to domains specialized for binding different receptors are widespread in eukaryotes too, including the previously unidentified actin cross-linking, ricin β-lectin and C-type lectin domains (See Supplementary Fig. S1). These observations suggest an even larger modularity for aerolysin-like PFTs, and that the receptor-binding region of these proteins is extremely variable.

**Figure 2:**
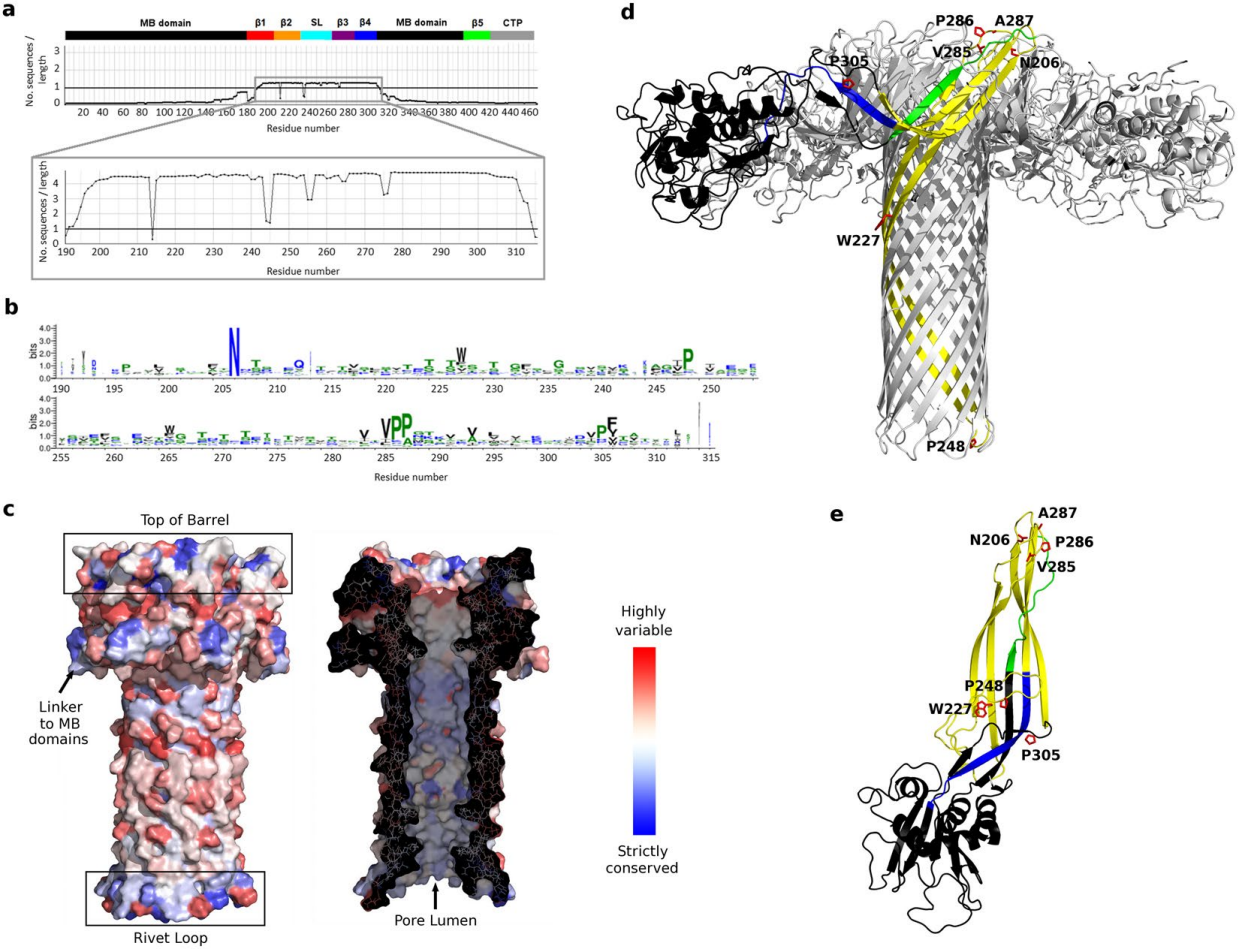
Sequence conservation within the aerolysin-like family. (**a**) Number of sequences counts for the full aerolysin sequence or only the segment 190–315, where a count is defined as a position with no gap on the alignment. Above the alignment, the aerolysin conserved structural regions are reported in bars colored according to Fig. 1. (**b**) Weblogo diagram of amino acid conservation computed from the alignment of the segment 190–315. (**c**) Amino acid variability from 1 (blue, strictly conserved) to 20 (red, highly variable), mapped on the aerolysin double β-barrel region. In the left, the exterior pore surface is shown and, in the right, the surface has been cut to show the pore lumen. (**d**) Aerolysin pore structure, with only one protomer colored according to: MB domain in black, segment 190–309 in yellow, segment 310–315 in blue, segment 408–421 (β5 strand) in green. The most conserved amino acids are shown as red sticks. The MB domains in the front have been removed to improve visualization. (**e**) Aerolysin soluble monomer structure, with the same coloring scheme as in (**d)**.

As a starting point towards the exploration of conserved and variable features in the aerolysin family, we generated an alignment from its full sequence (PDB entry 1PRE) using the HHblits program through the Gremlin webserver^23,24^. This provided a filtered, low-redundancy alignment with sequences containing 25% of gaps at most; in addition, Gremlin returned coevolution patterns that we analyzed later on in this work. The resulting alignment contained only 43 sequences, with the segment of higher sequence counts mapping to residues 190-315, *i.e*. not covering the full PF domain but limited to the inner β-barrel and the outer β-barrel region of the pore structure (residues 190–300, yellow in Fig. 2d) followed by 15 additional residues (blue strand in Fig. 2d) that connect the PF and MB domains. Strand β5, which represents the third strand of the outer β-barrel (green strand in Fig. 2d), is not captured by the retrieved alignment, most likely because of its short size and highly variable location downstream of strand β4.

Focusing on the DBB region, we built a new alignment using as query sequence only the segment spanning residues 190–315 of aerolysin. This produced an alignment of 559 sequences after filtering, which covered quite smoothly the query segment (Fig. 2a). From this alignment we could quantify amino acid conservation and map it on the available structures, finding out that the top of the pore, the linker to the MB domain, the rivet loop and the pore lumen are the most conserved elements (Fig. 2c). A weblogo visualization of the alignment (Fig. 2b) further pointed at the most conserved positions corresponding to residues N206, W227, P248, V285-A287 and P305, in the aerolysin numbering. In the pore structure, N206 and V285-A287 are found on the top of the DBB; P248 is located at the β-turn of the rivet loop; and W227 is in the exterior surface of the inner β-barrel (Fig. 2d). Such position does not explain an important role for W227 that justifies its conservation; however, in the monomer and prepore structures, W227 is located between strands β2-β3 and the stem loop, suggesting a role in the refolding mechanism that leads to pore formation (Fig. 2e). Finally, P305 is located in the middle of a β-strand in the monomer and prepore structures, which is expected to introduce stress given the strand-breaking nature of proline. In fact, in the pore structure this strand region refolds into a β-turn (Fig. 2d,e).

In the former alignment, the sequences of ETX, parasporin-2 and LSL were retrieved, but not those of Dln1, lysenin, CPE or HA3. Therefore, we proceeded as above but using the core barrel sequences of these last 4 proteins, and explored the overlap between the obtained alignments. We retrieved 380 Dln1-like sequences, 43% of which overlap with those retrieved from aerolysin’s sequence, but only 4 lysenin-like sequences (all belonging to the same organism as the query sequence), 9 CPE-like sequences and 9 HA3-like sequences. Four of the sequences retrieved from CPE and HA3 sequences are the same protein, an overlap that reflects the structural similarity of these two proteins; but none of these protein sequences was retrieved from the sequences of either aerolysin, Dln or lysenin, and *vice versa*, which most likely reflects their very different monomer structures (Fig. 1c,d).

### Structural and functional insights from the DBB core sequence alignment

Using the core alignment of aerolysin DBB, we analyzed the 190–315 segment in terms of physicochemical and coevolution constraints to variability, interpreted in the context of the available monomer, prepore and pore aerolysin structures.

**Figure 3:**
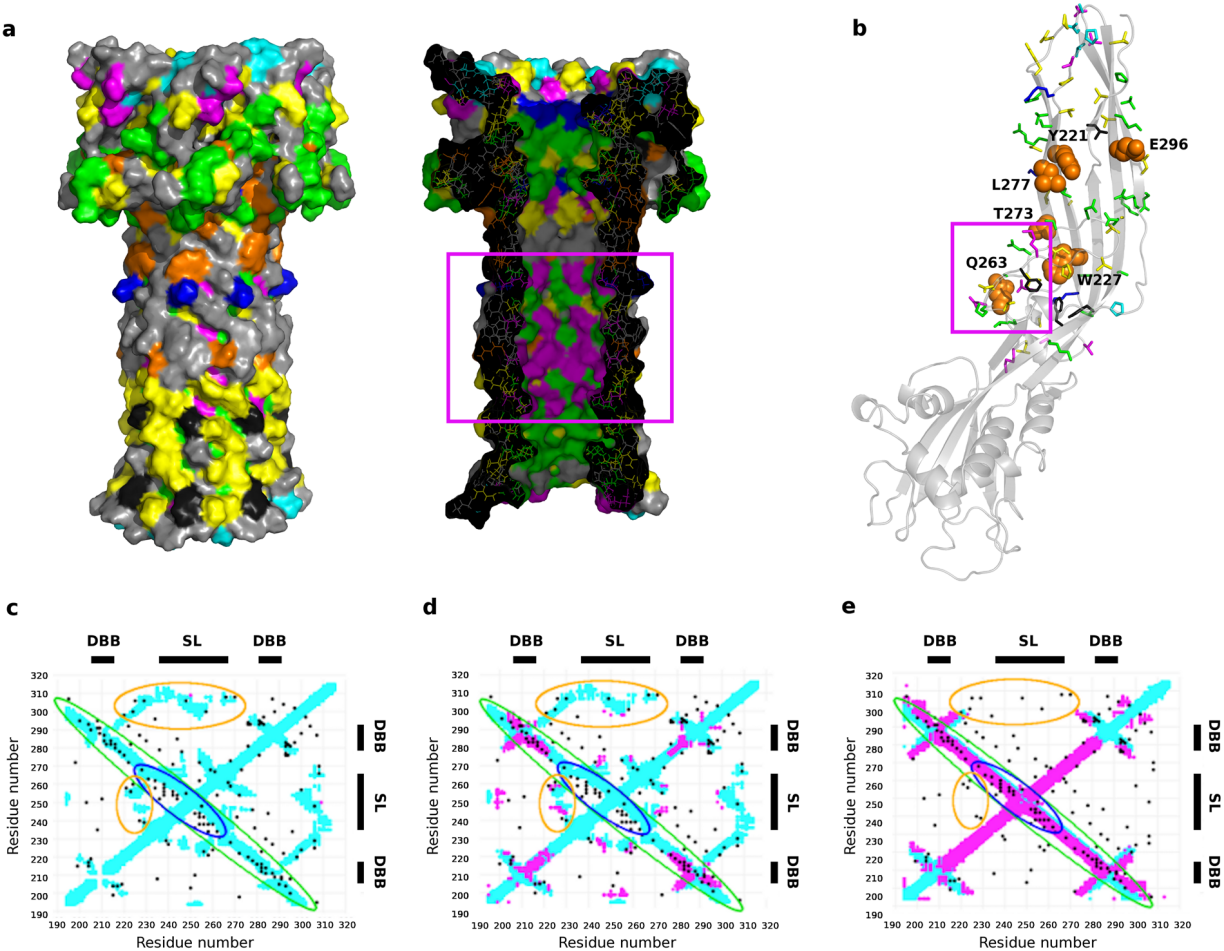
Physicochemical and coevolution constraints to variability within the aerolysin-like family. (**a**,**b**) Conserved amino acid physiochemical properties, mapped on the double β-barrel region of the pore structure in (**a)** or in the soluble monomer in (**b)**: magenta = preference for small volumes (suggests flexibility is required), green = preference for hydrophilic amino acids, blue = preference for soluble amino acids, black = preference for hydrophobic amino acids, yellow = preference for metabolically inexpensive amino acids, orange = distributions shape by backbone conformational descriptors (labeled and shown as spheres in **b**). In cyan, residues highly conserved from Fig. 2. Magenta boxes show the residues of the hinge between the stem loop and strands β2-β3. (**c–e**) Coevolution-predicted contacts above the noise level for the aerolysin-like family, calculated from the sequence alignment retrieved using as target the aerolysin sequence from 190–315, and compared to contact maps for aerolysin in the monomer (**c**) prepore (**d**) and pore structures (**e**). Black dots mark pairs of coevolving residues, the contact map in light blue corresponds to intra-monomer contacts from the structures, and the contact map in magenta corresponds to inter-monomer contacts. The double β-barrel (DBB) and stem loop (SL) regions are represented on the sequence. The coevolution patterns mentioned within the text are highlighted with colored ellipses.

Physicochemical constraints to variation reflect functional roles of individual residues by highlighting which amino acid properties (e.g., small or large volumes, hydrophobicity or hydrophilicity, etc.) are most favored at each position of the alignment, an analysis implemented in our webserver PsychoProt^25,26^. On the alignment generated from the sequence segment 190–315, PsychoProt returned fits for 83 residues (~66% of the analyzed segment) (Fig. 3a,b). As expected, the analysis indicates a preference for hydrophobic amino acids in the transmembrane region and in some positions between the two concentric β-barrels (black and also yellow in Fig. 3a,b, as most residues preferring metabolically inexpensive amino acids are also hydrophobic). Preference for soluble and hydrophilic residues was found in the exposed regions of the DBB, as well as in the pore lumen, consistent with their polar natures (green and blue in Fig. 3a,b). A series of sites where small (i.e. highly flexible) amino acids are preferred map to the top of the pore structure (magenta in Fig. 3a,b). There is also a region with preference for small amino acids inside the pore lumen, whose importance can be understood only in the monomer and prepore conformations, where these residues locate on the loops between strands β2-β3 and the stem loop, a hinge region that requires flexibility for the release of the stem loop during pore formation (magenta boxes in Fig. 3a,b). More interestingly, amino acid preferences at residues 227 (Trp in aerolysin) and 221 (Tyr in aerolysin) are best explained by descriptors about backbone conformation (orange in Fig. 3a,b), which suggests they could be important for conformational changes along the refolding pathway. In line with this, mutation Y221G is known to block the transition from prepore to pore state^27^. Other residues whose amino acid preferences are also shaped by backbone conformational descriptors are Q263, T273, L277 and E296.

The second stage of this analysis is based on residue-residue coevolution provided by the Gremlin server after alignment generation^23^. The rationale for such analysis is that patterns of coevolving residues encode information about the structure of the folded state and of alternative conformations^28–31^. The coevolution scores from the alignment of the 190-315 segment pick up a strong pattern for the long β-hairpin that forms the inner barrel in the pore structure, running antidiagonally from residue ~210 to 290 (strands β1, β2 and stem loop) (green ellipse in Fig. 3e). Part of this antidiagonal pattern also matches the contacts in the monomer and prepore structures (green ellipse in Fig. 3c,d); however, couplings for the segment ~235–265 (stem loop) do not match, being shifted by 5-10 residues (blue ellipse in Fig. 3c–e). The match between coevolution couplings and contact maps of the 235–265 segment only in the pore structure is consistent with the sliding motion required to form the barrel upon refolding^20,32^, and suggests tighter interactions in the pore structure. On the other hand, several weaker contacts are better satisfied in the monomer and prepore structures than in the pore structure. Among them, contacts in the two areas defined by residues ~235–265 and ~220–230, and by residues ~300–310 and ~220–270 (orange ellipses in Fig. 3c–e) would correspond to interactions meant to keep the stem loop bound to the β-strands before its release for pore formation. These observations suggest that the stem loop is trapped in a frustrated, metastable conformation, which relaxes only upon full assembly of the pore.

### Dynamic features of the monomeric toxins

The sequence analyses hinted at functionally relevant elements of the DBB and stem loop, and proposed roles based on conformational changes for some of these residues. These analyses, however, do not inform about the likely directions and magnitudes of the different motions. To gain a deeper mechanical insight, we therefore used submicrosecond atomistic molecular dynamics (MD) to sample the conformational landscape of aerolysin-like active monomers. We simulated the monomeric form of wild type aerolysin, an aerolysin mutant (Y221G) that arrests in the prepore state upon oligomerization, and four related PFTs (ETX, ETX H162A mutant, parasporin-2 and LSL), as a means to explore conservation and variability of dynamic features as related with previous bioinformatics analyses (See Supplementary Table S1 for a list about all simulations performed and Supplementary Fig. S2 for the RMSD plots).

**Figure 4:**
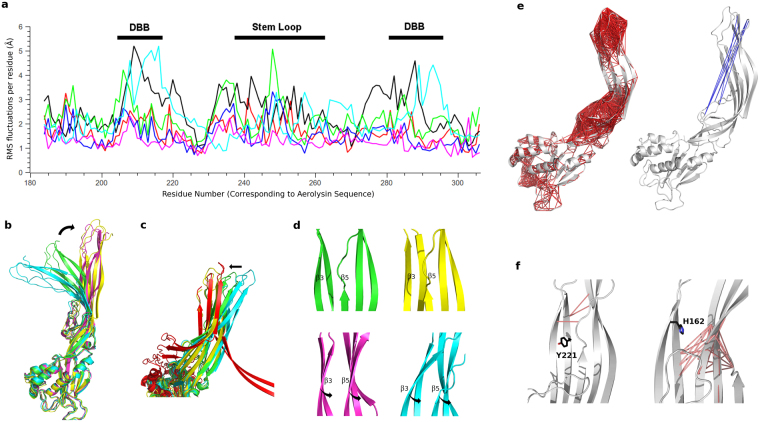
Conserved dynamics of the aerolysin-like proteins. (**a**) RMS fluctuations per residue in the PF region (corresponding to residues 195–310 of aerolysin), during the MD trajectory of each toxin: aerolysin wt (red), aerolysin Y221G (black), ETX wt (green), ETX H162A (blue), parasporin-2 (magenta) and LSL (cyan). (**b–d**) Three main monomer motions (PCs) as obtained from the PCA, superposed with the monomer (green), prepore (magenta) and pore (red) aerolysin structures. The three main motions are represented by the two extreme projections along the MD trajectory on the average structure of aerolysin Y221G (shown in yellow and cyan): PC1 (**b**); PC2 (**c**), in a look-up of domain 4, showing that one of the extreme projections superposes with domain 4 in the pore state, while the other resembles the soluble state; and PC3 (**d**), in a look-up of domain 3, showing the motion of strands β3 and β5 that lead to their parallel disposition in the prepore oligomer. (**e**) Correlated (left) and anti-correlated (right) residues depicted with red and blue lines, respectively, as calculated from the aerolysin wt MD trajectory. (**f**) Decreased cross-correlations between the wt and the mutant forms for aerolysin (left) and ETX (right). The aromatic amino acid that holds the mutation is shown.

During the MD simulations, all proteins presented the strongest fluctuations at the DBB and stem loop regions (Fig. 4a), even though the magnitude of the motions differed between toxins, with aerolysin Y221G showing the highest RMSD values while parasporin-2 appeared as a much more rigid protein. To identify the key protein motions, we performed principal component analysis (PCA) on the MD trajectories, visually observing that the 3 first principal components (PCs), that is, the three largest amplitude motions, were common to all proteins. In an attempt to understand the role of these 3 dominant motions, we compared the extreme conformations obtained projecting the aerolysin Y221G MD simulation onto each of the three first PCs, with the structures of aerolysin in the soluble, prepore and pore states. We used aerolysin Y221G and not the wt because the mutant form had explored a much broader conformational space during the MD simulation. From this study it became clear that PC1 is related to a distancing observed between the MB and the PF regions, leading to the prepore aerolysin Y221G conformation (Fig. 4b). The hinge region for this motion is located around residues L277-Y221-E296, and at the stem loop, near residue E254 (See Supplementary Fig. S3a). Interestingly, the variability observed in the alignments at the three first positions was explained by descriptors about backbone conformation in our PsychoProt analysis, consistent with roles for these residues in acting as a hinge that controls conformational changes. Also PC3 explains a conformational change between the soluble and the prepore states, namely a disposition of strands β2-β3 parallel to strands β1-β4-β5 (Fig. 4d). The hinge motion for PC3 occurs in the same region as PC1 (See Supplementary Fig. S3c). Finally, PC2 fits with a change in domain 4 related to the formation of the two concentric β-barrel fold in the aerolysin pore structure (Fig. 4c). In this case, the hinge region is seen around residues Y221 and T273, both related to descriptors about backbone conformation (See Supplementary Fig. S3b). Finally, we performed Normal Mode Analysis (NMA) of lysenin and CPE monomers, observing the conservation of these three main motions even in the more structurally divergent aerolysin-like toxins.

Subsequently, we calculated the cross-correlation coefficients between pairs of residues along the MD trajectory (Fig. 4e for aerolysin wt and Supplementary Fig. S4 for the other toxins), which helps identifying correlated collective motions (positive or negative values for motions occurring in the same or opposite direction, respectively)^33^. The results showed that, for all toxins, the strongest correlations occur within amino acids inside the DBB and the stem loop. More interesting are the anti-correlations, being the strongest performed between the DBB region and either the stem loop (in aerolysin) or the strands behind the stem loop (in ETX and parasporin-2), corresponding to the motion previously described in PC1 (Fig. 4b). In this analysis, however, we were also able to detect the amino acids performing the strongest anti-correlations, which, for aerolysin wt, are P181, K246 and W247 at the stem loop, with R288, S289, K290, P421 and L422 at the DBB. Moreover, calculating the difference between the cross-correlation profiles of the wt and the mutant forms, we could get some clues about the reason why both aerolysin Y221G and ETX H162A mutants (H149A according to the numbering of Oyston *et al*.^34^) present none or decreased pore formation in spite of retaining its ability to oligomerize^27,34^. Aerolysin Y221 is located in strand β2, while ETX H162 is located in a similar position (corresponding to aerolysin residue L277), but in strand β3. Both for aerolysin and ETX, the strongest decrease in cross-correlations on the mutant proteins was observed between strands β2-β3 and strands β1-β4-β5 (Fig. 4f), suggesting that these aromatic amino acids could be involved in the release of strands β2-β3 to form the pore. Interestingly, parasporin-2 and LSL also present aromatic residues either in the position of aerolysin Y221 or ETX H162. Moreover, both positions were explained by descriptors about backbone conformation as presented above.

### Dynamic features of the aerolysin oligomer structures

We finally studied, using NMA and atomistic MD, the dynamics of the aerolysin wt prepore and pore, with the aim of unveiling how the prepore complex might refold into the final pore in the last step of the pore forming process. A model of the wt prepore was created from the prepore structure, which corresponds to the Y221G mutant, as described in the methods section. In order to explore how the presence of the lipid bilayer could influence the prepore dynamics, we performed two MD replica of 130 ns each, one for the prepore in water, and another for the prepore within interacting distance (4–6 Å) to a lipid bilayer. To study the pore dynamics, we used a model we recently presented based on the quasi-pore structure^20^, for which we performed 250 ns of unrestrained atomistic MD of the oligomer, with the TM barrel inserted on the membrane (See Supplementary Table S1 for a list about all simulations performed and Supplementary Fig. S2 for the RMSD plots).

Both the prepore and the pore structures show similar interaction with the lipid bilayer during MD, where, through a balancing motion of the whole oligomer, as well as an up-down motion of the MB domains, only a few protomers at a time lie close to the membrane. The residues interacting with the lipids are the same in both simulations, and they include W45, H132, Y162, R163, R336, W324 and H332. It is important to note that during MD of the pore structure, we observed the MB domains shifting down from their initial position to interact with the membrane, in this way leaving the DBB fully exposed to the solvent (See Supplementary Fig. S5). This conformation seems in agreement with some low-resolution images obtained by atomic force microscopy (AFM)^35^, which show that the aerolysin pore structure would not be completely flat on the membrane surface but creates a conic surface with a star-shaped topography. This architecture is consistent with the membrane-binding domains getting closer to the membrane as found in our MD simulations (confront Supplementary Information Fig. S5 with Fig. 3 of He *et al*.^35^).

**Figure 5:**
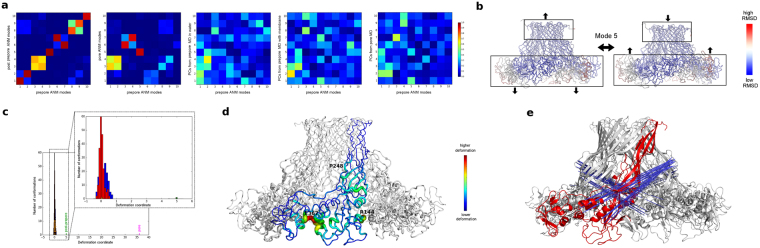
Aerolysin oligomers dynamics. (**a**) Overlap coefficients between the prepore normal modes and, from left to right, the post-prepore modes, the pore modes, and the PCs calculated from the MD trajectories of the prepore in water, the prepore in contact with a lipid bilayer, and the pore. (**b**) Mode 5 of the NMA calculated from the prepore model, showing a piston-like motion. (**c**) Projection onto mode 5 of the prepore MD trajectories (red and blue for simulation in absence and presence of a lipid bilayer, respectively), together with the prepore (orange), post-prepore (green) and pore (magenta) structures. (**d**) Hinge regions for mode 5, with only one protomer colored, and showing residues P248, R144 and E367. (**e**) Anti-correlations between residues on mode 5, with only one protomer colored in red.

To get a better insight into the main oligomer motions, we performed NMA of the prepore, post-prepore and pore structures. Through an overlap metric^36^, we observed that the five first modes predicted for the prepore are conserved in all three structures, even if their order (according to their relative amplitude) changes in the pore state (Fig. 5a). For the prepore structure, the two first modes alone account for ~50% of the overall dynamics (considering the first 10 modes) (See Supplementary Fig. S6a). They correspond to motions located mainly on the MB domains, either distancing-approaching from the tetramer axis, or from a hypothetical membrane (See Supplementary Fig. S6b), happening out-of-phase in the different protomers, and resembling the previously described balancing behavior. To better correlate these predicted motions with the dynamics observed on the MD simulations, we compared the prepore normal modes with the PCs of each MD through an overlap metric (Fig. 5a). In general, modes 1 and 2 present overlap with several PCs in all simulations. However, we observed an interesting fact: while for the MD of the prepore in water the first PC shows a better overlap with mode 1, for the MD simulations in the presence of a lipid bilayer, both for prepore and pore, the best overlap of PC1 is observed with mode 5. This mode, which was responsible of barely 6% of the prepore NMA variance (considering the first 10 modes), is therefore related to the highest amplitude motion in the membrane simulations, which recovered ~30% and ~50% of the total dynamics for prepore and pore, respectively (See Supplementary Fig. S6a). In a visual inspection of mode 5, we observe the piston-like motion expected to be the path to TM barrel insertion in the membrane^20^ (Fig. 5b).

Calculating the vector that explains the deformation or transition between two conformational states, and performing the overlap metric with some predicted modes it is possible to detect which of those modes (therefore motions) are responsible for the studied conformational change. When we performed this analysis comparing the prepore NMA modes with a vector calculated between the prepore and the pore structures, we observed a good overlap (~40%) just for mode 5 (See Supplementary Fig. S6c). Further, projecting the two prepore MD trajectories onto mode 5, together with the structures of the prepore, post-prepore and pore states, we observed that, in the presence of the lipid bilayer, the protein explores conformations in the direction of the post-prepore and pore states (Fig. 5c). Having detected the mode responsible for the transition from the prepore to the pore conformation, it is possible to study the residues most involved in that motion. The main hinge is located on the MB domain (mainly residues R144 and G366-V368), and extends through strands β1-β4-β5 up to the stem loop (around P248) (Fig. 5d). Concerning the cross-correlations, the biggest values are found within the DBB region and within the MB domains, while the strongest anti-correlations happen between the MB domain of one protomer (amino acids S133-Y136 and Q379-N384) and the region of strands β1-β4 that lies close to the stem loop of the adjacent protomer (amino acids D182-G187, so as Y304 and P305) (Fig. 5e).

Finally, we performed NMA of the prepore oligomer of HA3, and of the pore structure of lysenin, observing the existence of the piston-like motion in both of them. It must however be stressed that, while for aerolysin (both for pre-pore and pore), this motion was predicted as the fifth lowest frequency mode, for HA3 prepore, it is predicted as the highest amplitude one, and for lysenin pore as the eighth lowest frequency mode.

## Discussion

**Figure 6:**
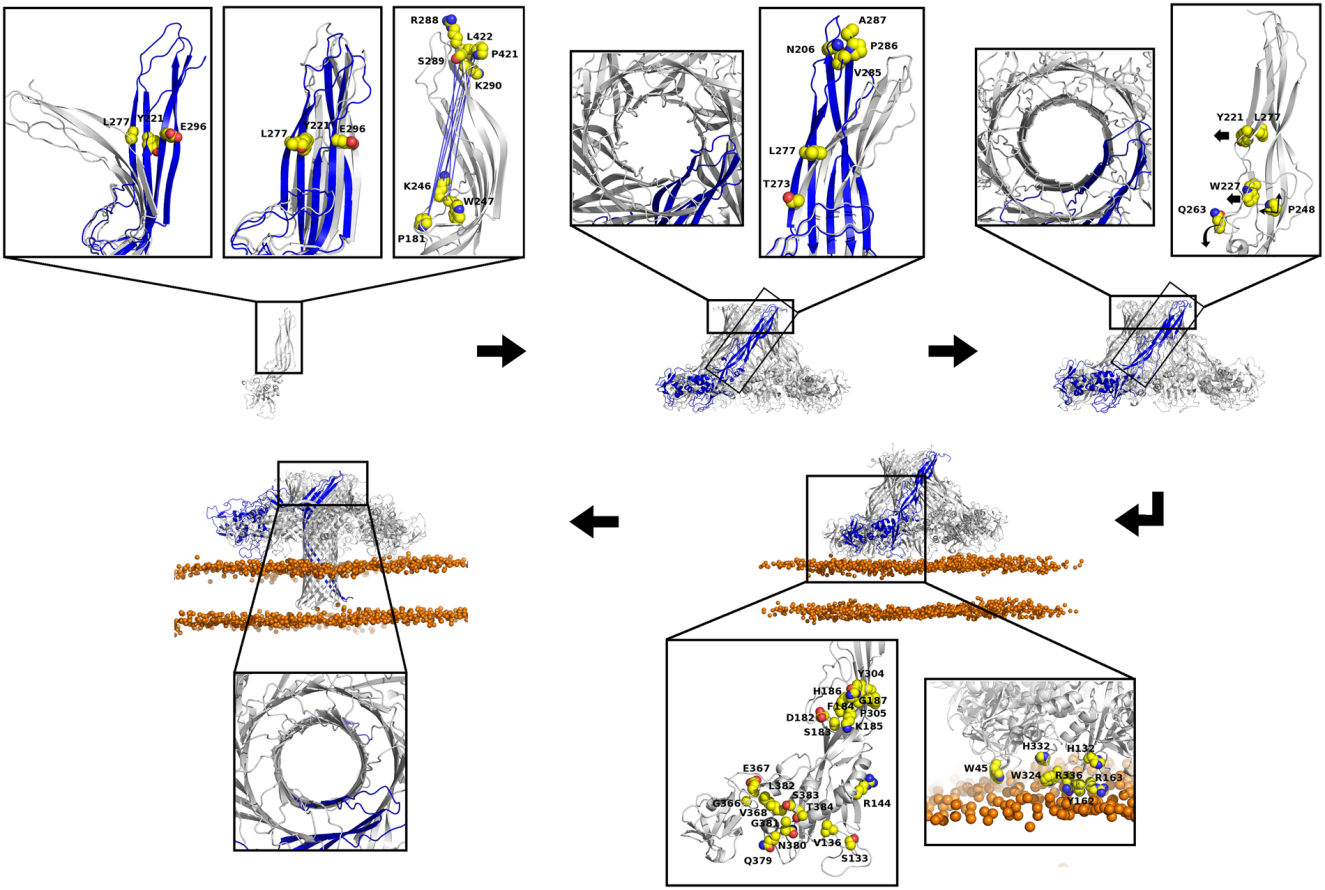
New insights into the pore forming process of aerolysin-like toxins. In the figure, the main steps leading to pore formation are highlighted: soluble monomer, soluble prepore and post-prepore oligomers, membrane-interacting oligomer and pore. For each step, the close-up images show, represented by spheres, the functionally relevant residues as suggested by our results, colored according to: carbon in yellow, oxygen in red, nitrogen in blue. When needed, black arrows are stressing the motions that may be induced by those residues. On the oligomers, only one protomer is colored in blue. On the representation of the PCA motions, only one extreme conformation is colored in blue. The lipid bilayer is represented showing only the phosphate ion as an orange sphere. On the close-ups of the first step, from left to right: motions described by PC1 and PC3, and the strongest anti-correlations during the aerolysin wt MD. On the second step, the close-ups show, from left to right, a partially formed DBB on the prepore, and the motion described by PC2. In the third step, the close-ups show a more perfect DBB formed on the post-prepore, and the main residues that may be involved in the release of the stem loop and strands β2-β3 to form the TM barrel. For the fourth step, the membrane interacting residues, so as those involved on the piston-like motion, are shown from right to left. Finally, on the close-up of the pore we can see the final DBB conformation.

Aerolysin-like proteins are being increasingly studied in the biotechnological field for their use in applications such as drug delivery to cancer cells or macromolecule sequencing. However, little is known about the pore forming mechanisms of these proteins, which hinder the research of new PFT-based strategies. To date, the pore structure of two aerolysin-like proteins (aerolysin itself and lysenin) have been published at atomic resolution, and, even if their pore stoichiometries were different (7 and 9 for aerolysin and lysenin, respectively), both present a novel fold constituted by two concentric β-barrels at the extracellular side. Moreover, the release of two other oligomer structures of aerolysin, arrested at the prepore and post-prepore states, makes this protein the best characterized isoform to get deep insights into the mechanism of pore formation. Here, we used structural information about the aerolysin-like proteins, together with sequence analysis tools, to map the amino acids that could be mostly involved in pore formation within this family of toxins (Fig. 6). Our results agree and explain the functional effects of several aerolysin mutations already reported by others, providing a richer mechanistic insight. In Supplementary Table S2, we present a summary of our results related to published experimental data, when available^27,32,37–39^.

Upon toxin activation, the first step in the pore formation process is the oligomerization into the prepore structure. MD of the active soluble proteins revealed two main motions that are related to oligomer conformation, here described as PC1 and PC3. The first one involves a distancing between the MB and the DBB regions, while the second represents a parallel arrangement of strands β2-β3 relative to strands β1-β4-β5. The hinge region for those motions is located around aerolysin amino acids L277, Y221 and E296, and at the stem loop, near E254. The three first residues were related to descriptors about backbone conformation on the physicochemical analysis of the aerolysin-like sequence alignment. Corresponding to PC1, we observed the strongest anti-correlations during the MD trajectories between the stem loop and the DBB region. For aerolysin, the main positions involved in those correlations were the stem loop amino acids P181, K246 and W247, and the DBB amino acids R288, S289, K290, P421 and L422.

After oligomerization, in the conformational path to pore formation, aerolysin goes through an intermediate step named as post-prepore, where the two rings of β-strands constituting the DBB fold are already disposed as a more symmetric double ring, adopting its final conformation on the pore. Our results suggest that the formation of this fold contributes to the transition from the prepore to the final pore state. First, four amino acids located on the upper loops of the DBB were found to be highly conserved within the family, namely, those corresponding to aerolysin amino acids N206, V285, P286 and A287. Second, during the MD simulation of the monomeric toxins, we observed a conserved motion located on this region, here described as PC2, which matched to a straighter disposition of the DBB region on the pore structure. The hinge for that motion was found around residues L277 and T273, both related to descriptors about backbone conformation. Finally, during the piston-like motion, which we reinforce here to be the responsible for TM barrel insertion, the strongest correlations were found within the DBB region (so as within the MB domains).

However, the formation of a perfect DBB fold is not the only responsible lead for pore formation. To form the TM barrel, the release of the stem loop is needed, followed by strands β2-β3. Q263, whose position was described by descriptors about backbone conformation, is located on the linker between the stem loop and strands β2-β3, suggesting a role as a rotation point on the stem loop release. As for the release of strands β2-β3, our results suggest a role for the positions corresponding to aerolysin amino acids Y221 and L277. We already know that the Y221G mutant oligomerizes but does not form pores^27^. Moreover, the position of L277 corresponds to H162 in the ETX toxin, whose mutant H162A has decreased pore formation^34^. From our MD simulations, we observed that both mutant proteins presented decreased cross-correlations between strands β2-β3 and strands β1-β4-β5, when compared to the wt forms, which may be the reason of their decreased activity. The release of the stem loop could also be assisted by W227, a conserved amino acid located adjacent to it, which was also related to backbone conformation descriptors. Finally, upon pore formation, the amino acid pairwise contacts of the stem loop suffer a sliding of six positions to get to the rivet loop conformation^32^. We believe that P248, a residue highly conserved within the family, could be responsible of creating a kink bringing about the residue sliding. Apart from its position in the middle of the rivet loop on the pore, P248 was found to act as part of the hinge for the piston-like motion.

Furthermore, we observed that the MB domains, and mainly its interactions with the membrane, are needed for TM barrel insertion in a piston-like manner, as this motion was only observed on the MD simulations of the oligomers in the presence of a lipid bilayer. Some residues of the MB domain were found to be especially involved in this motion, as R144 and G366-V368, where the main hinge is located, or amino acids S133-Y136 and Q379-N384, which perform the strongest anti-correlations observed for this motion. The partners for those correlations were some amino acids located at or around the stem loop, namely, D182-G187, Y304 and P305. This last amino acid (P305), which is highly conserved in the family, is located in a β-strand that suffers a conformational change creating a β-turn upon pore formation. These facts, altogether, suggest that P305 could be important in the vertical collapse of the MB domains observed in the pore structure of both lysenin and aerolysin. Finally, according to our MD simulations, the amino acids most involved on interactions with lipids are W45, H132, Y162, R163, R336, W324 and H332.

Altogether, this work provides a deeper mechanistic insight into the pore forming process within this family of toxins, revealing the motions related to the main conformational changes, and suggesting roles for several amino acids. Our results show that the DBB is not only the most conserved region on this family, but also a mechanistically important fold, whose arrangements drive to pore formation. On the other hand, the MB domains are the most variable regions, which do not mean a less important role on the activity, as their interaction with lipids and their correlated motions with the PF region are necessary for piston-like TM barrel insertion. Finally, we show that the functionally relevant patterns found on aerolysin are conserved across the family, which suggests that all these proteins probably share similar pore-formation processes. Moreover, the fact that the lysenin, CPE and HA3 sequences were not retrieved during our sequence searches using aerolysin or Dln1 as queries, suggests that more proteins as today believed may belong to the aerolysin-like family, and will share similar oligomer structures, with a double β-barrel fold.

## Methods

### Setup of Molecular Dynamics Simulations

For the MD of the toxins monomeric active form, the following PDB entries were used, from which only one monomer was taken, and, if needed, the residues known to be cleaved for toxin activation were deleted: aerolysin wt^19^ (PDB entry 1PRE) and aerolysin Y221G^40^ ((PDB entry 3C0N, deleting residues 440–470 to obtain the active forms^41^); ETX wt^7^ (PDB entry 1UYJ) and ETX H162A^42^ (PDB entry 3ZJX), deleting residues 250–282 to obtain the active forms^43^); LSL^44^ (PDB entry 2Y9F); parasporin-2^8^ (PDB entry 2ZTB); and lysenin^10^ (PDB entry 3ZX7). Moreover, as in previous studies by our group^40^, domain 1 of aerolysin was deleted in order to reduce the computation time, as it is known to act as an independent folding unit^45^. If needed, missing side chains were added with the Modeller program version 9.13^46^. All of the proteins were solvated in a rectangular box of TIP3P water and neutralized by NaCl. Simulations were run using the GROMACS software version 4.8^47,48^, with the Amber99SB force field^49^, the SHAKE algorithm on all the bonds between hydrogen and heavy atoms, and Particle-Mesh Ewald, treating the electrostatic interactions in periodic boundary conditions. We chose an integration step of 2 fs. The temperature has been controlled by means of Langevin forces, using a damping constant of 1 ps^−1^. 800 ns of simulation were run for each protein.

For the prepore molecular dynamics simulations, we first created a model for the wild-type form using as template the prepore structure of aerolysin Y221G^20^ (PDB entry 5JZH), with the Modeller program version 9.13^46^, following a rigid procedure (without Simulated Annealing optimization). The system was then simulated for 180 ns using the GROMACS software version 4.8^47,48^, with a similar procedure as that used for the monomeric toxins. The conformation closest to the average of the production run (last 100 ns) was similar to the prepore Y221G structure. This conformation was further used as input to build up two new simulation systems, one of the wt prepore in water, and a second one of the prepore wt in the presence of a lipid bilayer. The topology for both the prepore and the 1-palmitoyl-2-oleoyl-sn-glycero-3-phosphocholine (POPC) membrane were obtained from the CHARMM-GUI web server^50^, using the charmm36 force field^51^. For the second simulation, the protein was manually located at interacting distance to the membrane (minimum distance was 4 Å). Afterwards, the systems were solvated in a rectangular box of TIP3P water and neutralized by NaCl. Simulations were run using the GROMACS software version 4.8^47,48^, with the charmm36 force field^51^. For the prepore without membrane, a similar procedure of that used for the monomer toxins was applied, to a final simulation time of 130 ns. For the prepore with membrane, the system was first equilibrated for 150 ns in a NVT ensemble, using a time step of 1 fs, the Berendsen thermostat^52^ for temperature coupling, and with both the protein and lipid atoms initially restrained. While the lipid restrains were gradually decreased to zero, the protein was kept fixed. Afterwards, it was simulated in the NPT ensemble, with the Nose-Hoover^53^ thermostat for temperature coupling and the Parrinello-Rahman^54^ for semi-isotropic pressure coupling, gradually releasing the protein restraints, and then leaving the free systems simulate for 130 ns.

For the wt pore simulation, an already published model based of the quasipore structure was used^20^. The protein-membrane system was built with the charmm-gui server^50^, using the membrane positioning as suggested by the PPM server^55^. The system was simulated using a similar procedure of that of the prepore with membrane, but simulating for a longer time of 250 ns.

### Analysis of main protein dynamics

Two methods were here used to explore the main dynamics of the studied proteins: Principal Components Analysis (PCA) and Normal Mode Analysis (NMA). PCA is a statistical method based on covariance analysis that uses an orthogonal transformation to convert a set of correlated variables into a reduced set of uncorrelated variables called principal components (PCs). In this way PCA maps high-dimensional data, MD trajectories in this work, into interesting low-frequency motions concerted over large number of atoms (first PCs, as opposed to higher-order PCs which reflect faster local fluctuations)^56^. The PCA procedure is based on the diagonalization of the covariance matrix, C_(i,j)_:

1 $${{C}}_{({\boldsymbol{i}},{\boldsymbol{j}})}=\langle {\rm{\Delta }}{{r}}_{{i}}\cdot {\rm{\Delta }}{{r}}_{{j}}\rangle $$

where Δr_i_ and Δr_j_ indicate the displacement vectors of atoms i and j, respectively, from their average positions. For the MD trajectories of the monomeric forms, PCA was performed on 800 frames containing backbone coordinates (one frame every 1 ns of simulation) using the *g_covar* and *g_anaeig* Gromacs tools^47,48^. The two extreme projections along the aerolysin Y221G MD trajectory on the average structure, calculated with the *g_anaeig* Gromacs tool, were used to compare with the aerolysin structures on the different states (monomer, prepore, post-prepore and quasi-pore). For the MD trajectories of the oligomers (prepore and pore), PCA was performed with the software Prody^36^, using 130 or 250 frames, respectively (one frame every 1 ns of simulation).

NMA is another well-established method to infer global protein motions, but in this case computed from a single (static) structure. The main concerted motions predicted by the NMA model make up a set of collective variables (normal modes) that are obtained from the second derivative matrix of the potential energy. In brief, NMA is a simple tool to quickly calculate the expected main global motions from a structure, whereas PCA extracts actual global motions from a MD trajectory. In the ideal scenario, NMA normal modes and PCA principal components should be similar. NMA was used instead of PCA in the lysenin and CPE monomers, as well as for the prepore of HA3 and the lysenin pore, as we did not perform MD on them. For aerolysin, NMA was used together with PCA in the analysis of the prepore and pore dynamics, to compare the main motions predicted for the proteins with the dynamics obtained in MD in presence and absence of a lipid bilayer. NMA of both the monomers and the oligomers were here performed by the Anisotropic Network Model (ANM)^57^ with the software Prody^36^, and the results were visualized with the VMD program^58^. The following structures were used for other aerolysin-like proteins: CPE monomer^17^ (PBD entry 2XH6), HA3 prepore^16^ (PDB entry 4EN6), lysenin pore^13^ (PDB entry 5GAQ). The hinge regions (flexible joints) were calculated by the software Bio3D^59^ as the regions of minima of mean square fluctuations for a given mode/PC.

Pairwise dynamic cross-correlation coefficients^60^ were calculated with the Bio3D software^59^, considering only Cα carbons, and results were plot in *The PyMOL Molecular Graphics System, Version 1.8 Schrödinger*, *LLC* with the Bio3D function *pymol.dccm*. Briefly, the cross-correlation matrices were calculated according to the following equation, giving values in the range from 1 (correlated) to −1 (anti-correlated):

2 $${C}_{(i,j)}=\frac{\langle {\rm{\Delta }}{r}_{i}\cdot {\rm{\Delta }}{r}_{j}\rangle }{{\langle {\rm{\Delta }}{r}_{i}^{2}\rangle }^{1/2}\cdot {\langle {\rm{\Delta }}{r}_{j}^{2}\rangle }^{1/2}}$$

The squared overlap (dot product) between two vectors was calculated with the software Prody^36^ to measure the alignment between the directions of a pair of given PCs/modes, presenting a value of 1 if they are identical and of 0 is they are orthogonal^61^.

3 $${O}_{i}(X)={M}_{i}X/\Vert {M}_{i}\Vert \Vert X\Vert $$

This procedure was applied to study the overlap between different sets of NMs, between NMs and PCs, and also between NMs and a vector representing the conformational change between two different structures.

### Data availability

The additional data that support the findings of this study are available from the corresponding author upon request.

## Electronic supplementary material

SI: Structural, physicochemical and dynamic features conserved within the aerolysin pore-forming toxin family
