# Supplementary material for: Structural, physicochemical and dynamic features conserved within the aerolysin pore-forming toxin family
Source: Sci Rep. 2017 Oct 24;7:13932. doi: 10.1038/s41598-017-13714-4 (PMC5654971; doi:10.1038/s41598-017-13714-4)
Supplement: Supplementary file 1 — SI: Structural, physicochemical and dynamic features conserved within the aerolysin pore-forming toxin family [file 41598_2017_13714_MOESM1_ESM.pdf]

Structural, physicochemical and dynamic features conserved within the aerolysin pore-forming toxin family

Nuria Cirauqui[1,2], Luciano A. Abriata[1], F. Gisou van der Goot[3] and Matteo Dal Peraro[1]*

[1]Institute of Bioengineering, School of Life Sciences, École Polytechnique Fédérale de Lausanne (EPFL) and Swiss Institute of Bioinformatics, 1015 Lausanne, Switzerland
[2]Department of Pharmaceutical Biotechnology, Universidade Federal do Rio de Janeiro, 21941-902 Rio de Janeiro, Brazil
[3]Global Health Institute, School of Life Sciences, École Polytechnique Fédérale de Lausanne (EPFL) and Swiss Institute of Bioinformatics, 1015 Lausanne, Switzerland

Supplementary Information

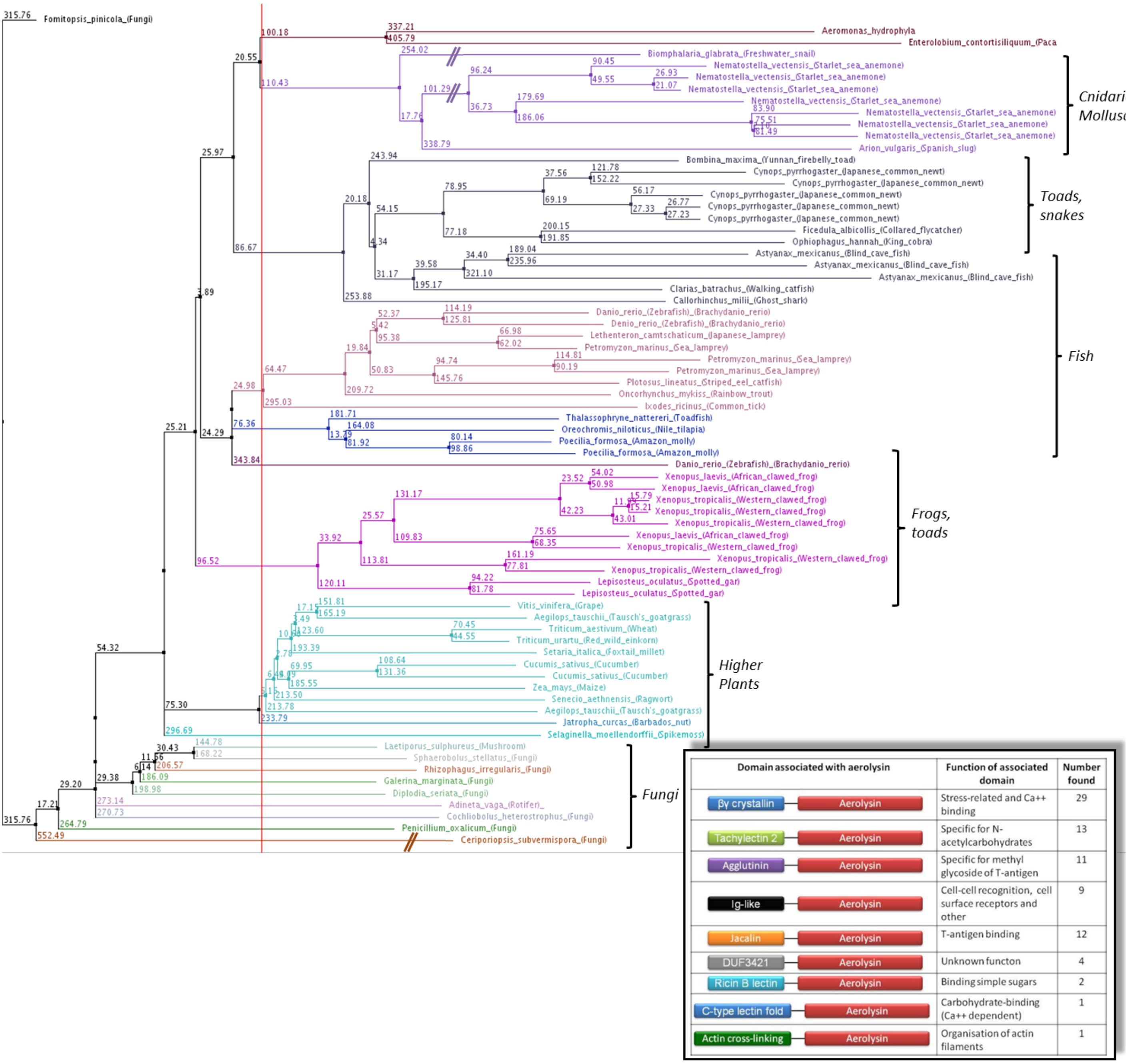

Domain associated with aerolysin	Function of associated domain	Number found
βγ crystallin – Aerolysin	Stress-related and Ca++ binding	29
Tachylectin 2 – Aerolysin	Specific for N-acetylcarbohydrates	13
Agglutinin – Aerolysin	Specific for methyl glycoside of T-antigen	11
Ig-like – Aerolysin	Cell-cell recognition, cell surface receptors and other	9
Jacalin – Aerolysin	T-antigen binding	12
DUF3421 – Aerolysin	Unknown functon	4
Ricin B lectin – Aerolysin	Binding simple sugars	2
C-type lectin fold – Aerolysin	Carbohydrate-binding (Ca++ dependent)	1
Actin cross-linking – Aerolysin	Organisation of actin filaments	1

Supplementary Figure S1. Phylogenetic tree of eukaryotic proteins containing an aerolysin domain. Previously known sequences of aerolysin proteins from different species (prokaryotic and eukaryotic) were blasted against UniProt. Sequences with identity above 30% and E-value threshold below 10^{-9} were retained. The phylogenetic tree was calculated using the neighbor-joining method with the PAM250 substitution matrix. Groups which appear to be evolutionary closer are presented in the same color and distance values between the branches are shown. Common names are given for groups of related species. Relative to the work by Szczesny *et al.*[1], the total number of new eukaryotic proteins with aerolysin domains is 55 and the number of new eukaryotic species with aerolysin domains is 30.

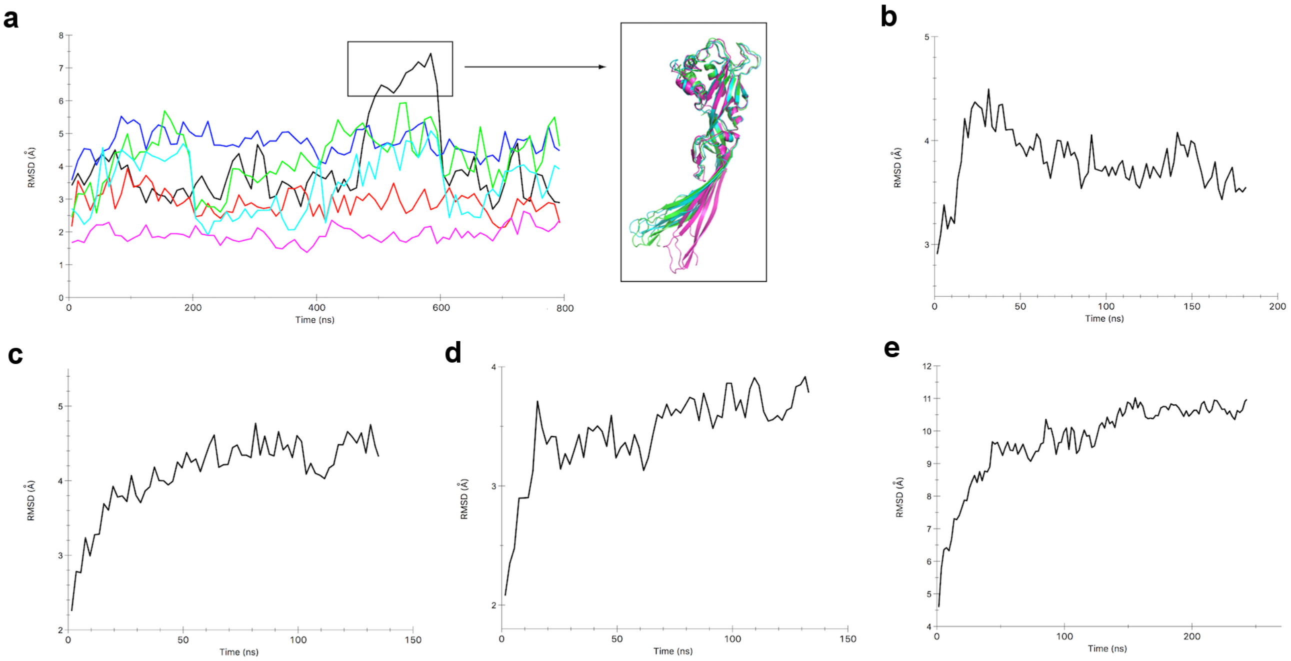

Supplementary Figure S2. Root Mean Square Deviation (RMSD) during the MD simulations. (**a**) Trajectory RMSD for the monomeric forms, taking as reference each initial conformation (corresponding x-ray structure), and averaged in 10 ns windows: aerolysin wt (red), aerolysin Y221G (black), ETX wt (green), ETX H162A (blue), parasporin-2 (magenta) and LSL (cyan). In the close-up, the aerolysin Y221G initial conformation (green), together with a conformation at 529 ns (magenta) and other at 634 ns (cyan) are represented as cartoons, showing that after a conformational change at 500-600 ns, aerolysin Y221G returns to its initial conformation. (**b**) Trajectory RMSD for the equilibration MD of the wt prepore model, taking as reference the initial model created from the x-ray structure of the aerolysin Y221G, and averaged in 2 ns windows. (**c**) Trajectory RMSD for the 130 ns of the wt prepore in water, taking as reference the average structure of the equilibration MD (**b**), and averaged in 2 ns windows. (**d**) Trajectory RMSD for the 130 ns of the wt prepore in presence of a lipid bilayer, taking as reference the average structure of the equilibration MD (**b**), and averaged in 2 ns windows. (**e**) Trajectory RMSD of the pore MD, taking as reference the model based on the quasi-pore structure[2], and averaged in 2 ns windows. The equilibration phase is not shown on the plots.

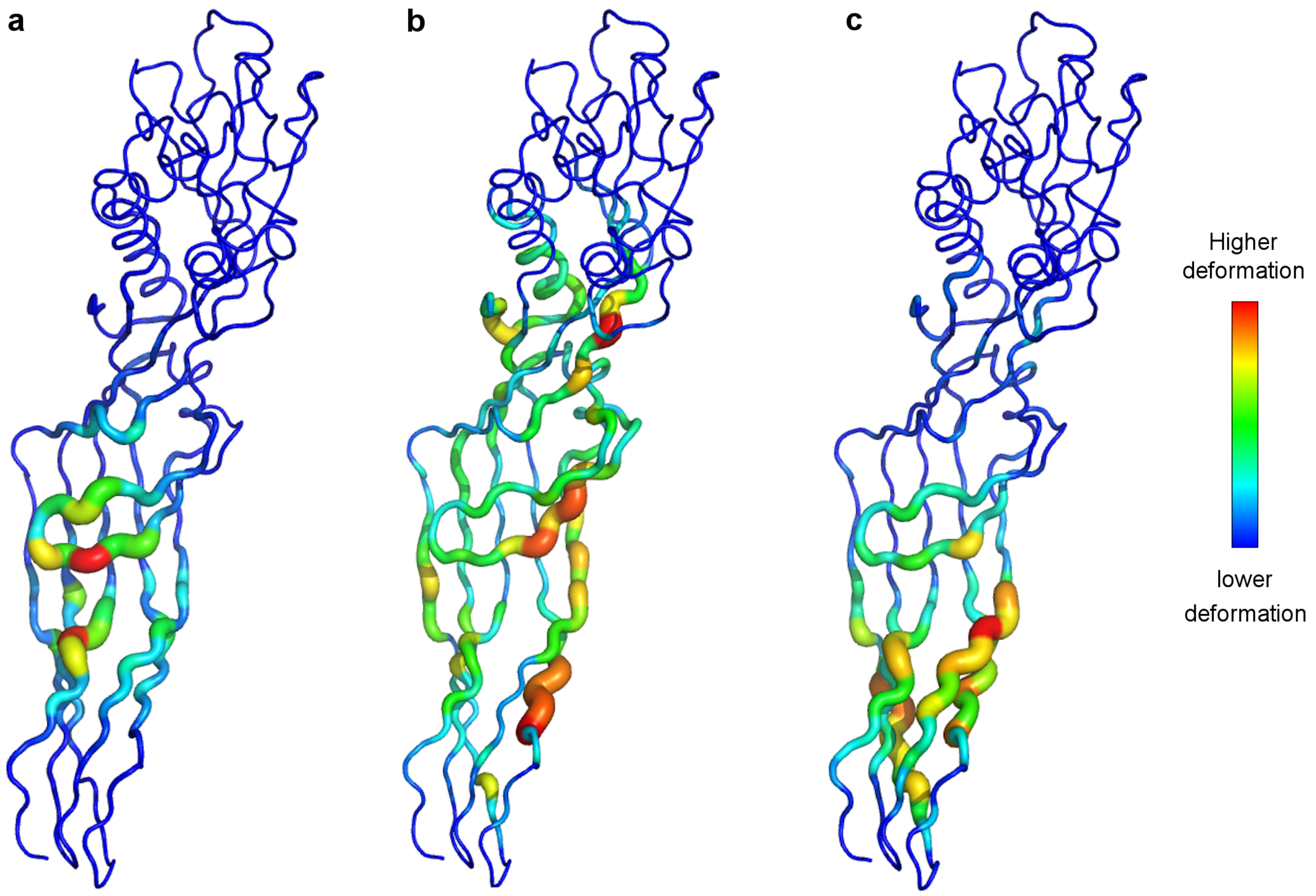

Supplementary Figure S3. Hinge regions of the first three principal components of the aerolysin-like monomers MD. Representation of the hinge residues for each of the three first PCs, shown here for the aerolysin Y221G toxin, where warmer colors indicate regions more involved in the deformation for that motion: (**a**) PC1, (**b**) PC2, (**c**) PC3.

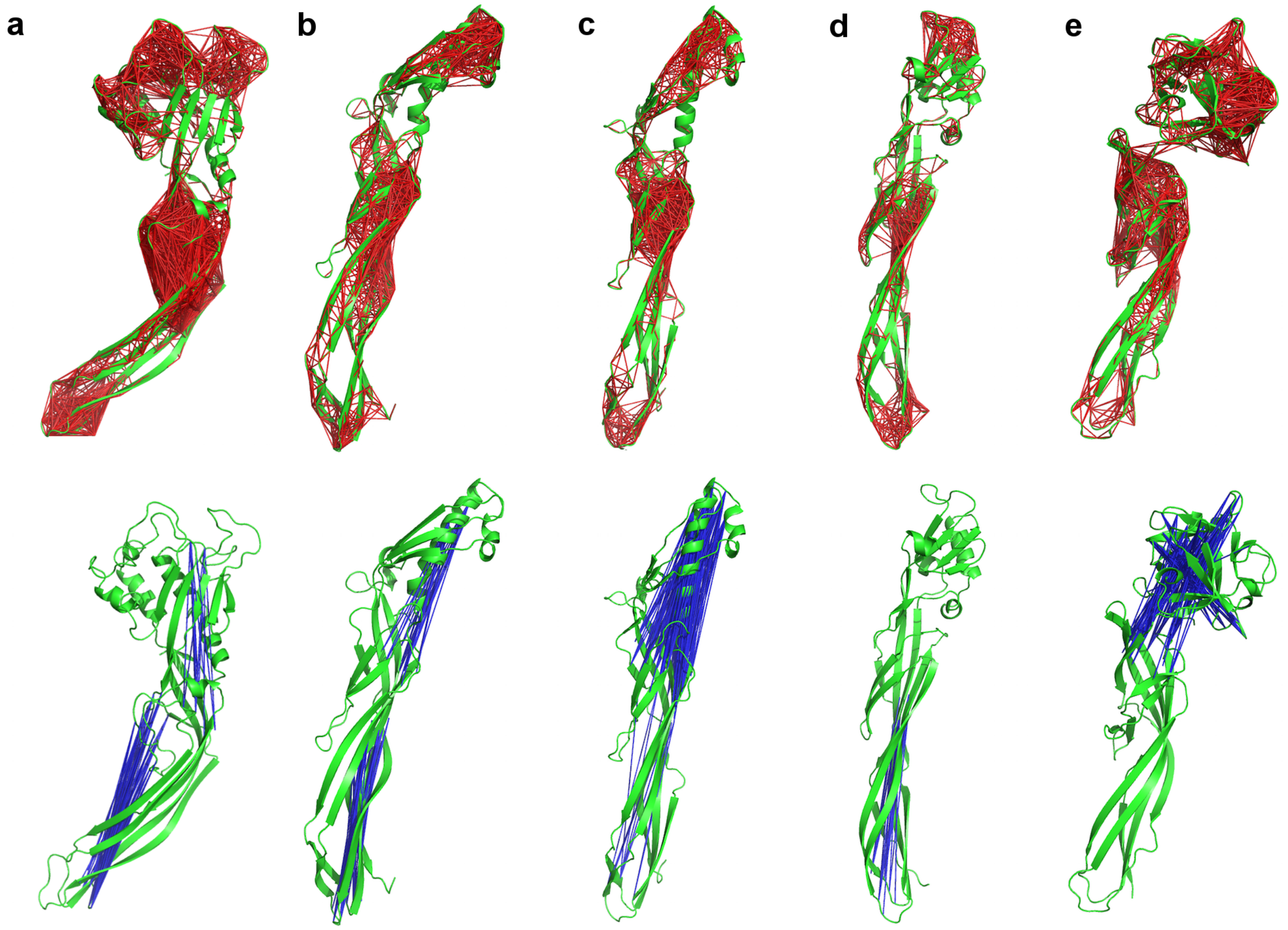

Supplementary Figure S4. Strongest cross-correlations found during the aerolysin-like monomers MD. Correlated (up) and anti-correlated (down) residues depicted with red and blue lines, respectively, as calculated from the MD trajectories of: (**a**) aerolysin Y221G, (**b**) ETX wt, (**c**) ETX H162A, (**d**) parasporin-2, (**e**) LSL.

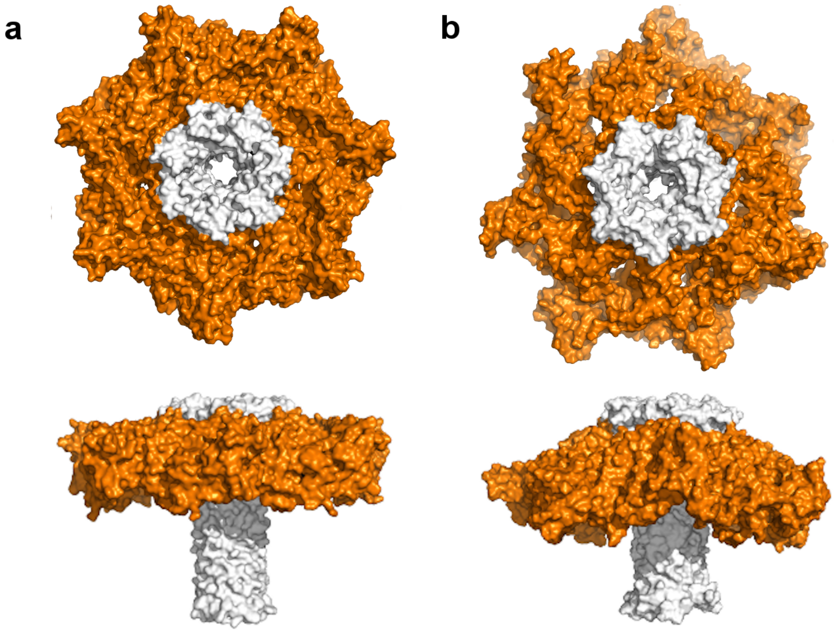

Supplementary Figure S5. New disposition of the MB domains of the aerolysin pore obtained upon MD simulation. (**a**) Molecular surface of the initial pore structure[2] (PDB entry 5JZW) from a view above (up) and parallel (down) the membrane plane. (**b**) Molecular surface of conformation closest to the production run average of the 250 ns of unrestrained atomistic MD, from a view above (up) and parallel (down) the membrane plane. For both **a** and **b**, the DBB and the TM barrel are shown in white, with domain 3 together with the MB domains in orange. The MD conformation (**b**) resembles the scan-size images of the crystalline aerolysin pore in He *et al.*, 2016[3], which showed a star-shaped topography of the pore (**c**, up) and a central ring greater in height than the radially projecting arms (**c**, down).

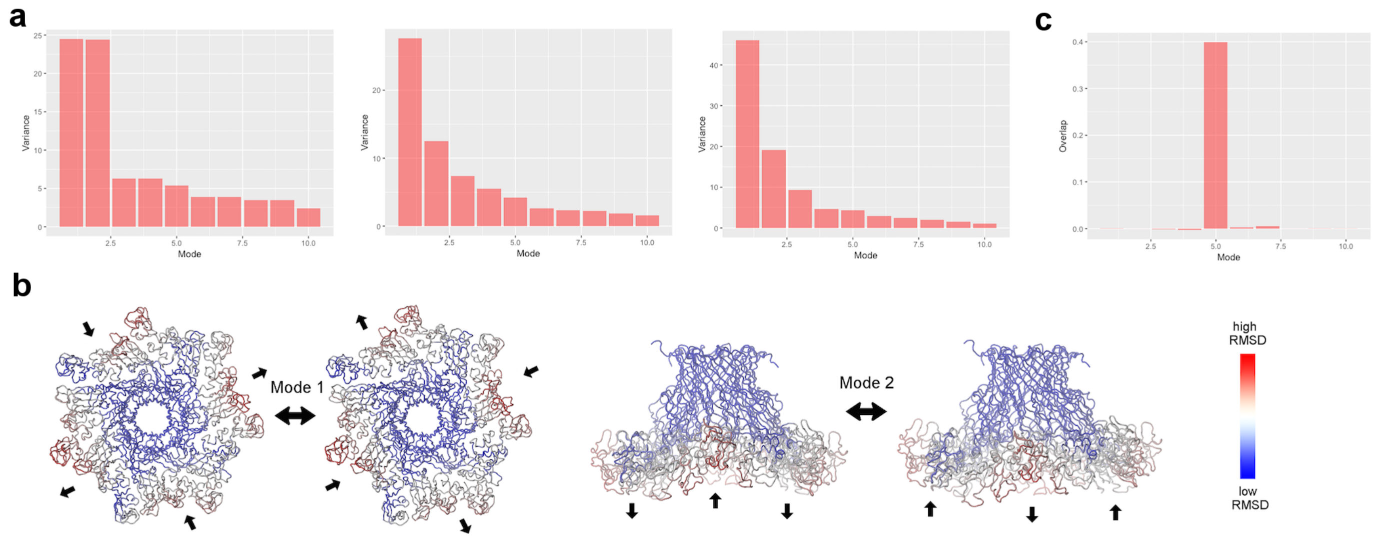

Supplementary Figure S6. **Supplementary data about the NMA and the PCA of both prepore and pore states of aerolysin.** (**a**) Percentage of variance calculated for the first 10 modes (or PCs) as obtained, from left to right, from: NMA of the aerolysin prepore structure, PCA of the MD trajectory of aerolysin prepore in presence of a lipid bilayer, PCA of the MD trajectory of aerolysin pore. (**b**) Representation of the motions described by modes 1 and 2 as calculated by NMA of the aerolysin prepore structure. (**c**) Ovelap between the prepore NMs and the vector calculated between the prepore and the pore structures.

Supplementary Table S1. Summary of the Molecular Dynamics (MD) simulations performed in this work.

Conformational state	Initial Structure	Simulation time (ns)
Monomer	Aerolysin wt	800
	Aerolysin Y221G	800
	ETX wt	800
	ETX H162A	800
	Parasporin-2	800
	LSL	800
Prepore	Aerolysin wt model (for equilibration purposes)	180
	Equilibrated model of aerolysin wt (in water)	130
	Equilibrated model of wt (with lipid membrane)	130
Pore	Aerolysin wt pore model	250

Supplementary Table S2. Comparison between the *in silico* results obtained in this work, and previously published mutational experiments of aerolysin toxin, together with an overview of the roles proposed here for each amino acid, according to Figure 6.

Amino acid[a]	*In silico* results[b]	Mutational data[c]	Proposed roles[d]
P248	Conserved residue	Decreased pore formation[4]	Creates a kink on the stem loop to help sliding during pore formation
N206, V285-A287	Conserved residues	No data found	Due to its location on the DBB loops, they could help in several steps, as the motion described in PC2 (creation of a more concentric DBB fold)
W227	Conserved residue, related to descriptors about backbone conformation	Mutation for other residues impairs protein secretion by bacteria, and therefore its role on pore-formation was not tested[5]	Pushes away the stem loop for its release during pore formation
Y221	Related to descriptors about backbone conformation, decreased cross-correlations between strands β2-β3 and strands β1-β4-β5 on the mutant Y221G, hinge on the motions related to oligomerization (PC1, PC3)	Oligomerizes in the prepore state but does not form pores[6]	Pushes away strands β2-β3 for its release during pore formation, also involved in oligomerization
Q263	Related to descriptors about backbone conformation	No data found	Rotation point on the stem loop release
T273	Related to descriptors about backbone conformation, hinge on the motion related DBB formation (PC2)	No data found	Acts as hinge on the motions described in PC2 (creation of a more concentric DBB fold)
E296	Related to descriptors about backbone conformation, hinge on the motions related to oligomerization (PC1, PC3)	No data found	Involved in oligomerization
L277	Related to descriptors about backbone conformation, hinge on the motions related to oligomerization (PC1, PC3). For ETX, a mutation on the	No data found	Pushes away strands β2-β3 for its release during pore formation, Involved in oligomerization

	corresponding position (H162A) presented decreased cross-correlations between strands β2-β3 and strands β1-β4-β5		
E254	Hinge on the motions related to oligomerization (PC1, PC3)	E254C: Slight reduction in hemolytic activity[4]	Involved in oligomerization
W247	Strong cross-correlations with the DBB region found during the MD of the active aerolysin monomer	W247C: No hemolytic activity[4]	May help in both oligomerization and pore-formation
K246	Strong cross-correlations with the DBB region found during the MD of the active aerolysin monomer	K246C: Slight reduction in hemolytic activity[4]	May help in both oligomerization and pore-formation
P181	Strong cross-correlations with the DBB region found during the MD of the active aerolysin monomer	No data found	May help in both oligomerization and pore-formation
R288-K290, L421, P422	Strong cross-correlations with the stem loop region found during the MD of the active aerolysin monomer	No data found	May help in both oligomerization and pore-formation
R144 G366-V368	Main hinge residues on the piston-like motion	No data found	Important for TM pore insertion on the membrane
P305	Conserved residue, strong cross-correlations on the piston-like motion	No data found	Creates a kink to move up MB domain during the piston-like motion
S133-Y136, Q379-N384, D182-G187, Y304	Strong cross-correlations on the piston-like motion	No data found	Important for TM pore insertion on the membrane
R336	Membrane interactions during prepore and pore MD	R336A does not form pores[7]	Important for membrane binding and therefore TM insertion
R163	Membrane interactions during prepore and pore MD	No data found	Important for membrane binding and therefore TM insertion
W324	Membrane interactions	W324A reduced pore	Important for membrane

	during prepore and pore MD	formation[8]	binding and therefore TM insertion
Y162	Membrane interactions during prepore and pore MD	Y162A small reduction in pore formation[8]	Important for membrane binding and therefore TM insertion
H132	Membrane interactions during prepore and pore MD	H132D, H132N: Impaired oligomerization[8]	Important for membrane binding and therefore TM insertion
W45, H332	Membrane interactions during prepore and pore MD	No data found	Important for membrane binding and therefore TM insertion

[a]Aerolysin amino acid

[b]*In silico* results obtained in this work for that amino acid

[c]Data found on the literature about aerolysin mutants on that position

[d]Roles proposed in this work for that amino acid based on our *in silico* results

References

1. Szczesny, P. *et al.* Extending the Aerolysin Family: From Bacteria to Vertebrates. *PLoS ONE* **6,** e20349 (2011).
2. Iacovache, I. *et al.* Cryo-EM structure of aerolysin variants reveals a novel protein fold and the pore-formation process. *Nat. Commun.* **7,** 12062 (2016).
3. He, J. *et al.* Single molecule atomic force microscopy of aerolysin pore complexes reveals unexpected star-shaped topography: Structural Studies of Aerolysin Pore Complexes. *J. Mol. Recognit.* **29,** 174–181 (2016).
4. Iacovache, I. *et al.* A rivet model for channel formation by aerolysin-like pore-forming toxins. *EMBO J.* **25,** 457–466 (2006).
5. Wong, K. R. & Buckley, J. T. Site-directed mutagenesis of a single tryptophan near the middle of the channel-forming toxin aerolysin inhibits its transfer across the outer membrane of Aeromonas salmonicida. *J. Biol. Chem.* **266,** 14451–14456 (1991).
6. Tsitrin, Y. *et al.* Conversion of a transmembrane to a water-soluble protein complex by a single point mutation. *Nat. Struct. Biol.* **9,** 729–733 (2002).
7. Osusky, M., Teschke, L., Wang, X., Wong, K. & Buckley, J. T. A Chimera of Interleukin 2 and a Binding Variant of Aerolysin Is Selectively Toxic to Cells Displaying the Interleukin 2 Receptor. *J. Biol. Chem.* **283,** 1572–1579 (2008).
8. MacKenzie, C. R., Hirama, T. & Buckley, J. T. Analysis of Receptor Binding by the Channel-forming Toxin Aerolysin Using Surface Plasmon Resonance. *J. Biol. Chem.* **274,** 22604–22609 (1999).
